# Biomechanical Testing of Suture Anchor Versus Transosseous Tunnel Technique for Quadriceps Tendon Repair Yields Similar Outcomes: A Systematic Review

**DOI:** 10.1016/j.asmr.2021.08.013

**Published:** 2021-09-30

**Authors:** John W. Belk, Adam Lindsay, Darby A. Houck, Jason L. Dragoo, James W. Genuario, Stephanie W. Mayer, Rachel M. Frank, Eric C. McCarty

**Affiliations:** University of Colorado School of Medicine, Department of Orthopaedics, University of Colorado, Aurora, Colorado, U.S.A.

## Abstract

**Purpose:**

To systematically review the literature to evaluate the biomechanical properties of the suture anchor (SA) versus transosseous tunnel (TO) techniques for quadriceps tendon (QT) repair.

**Methods:**

A systematic review was performed by searching PubMed, the Cochrane Library, and Embase using PRISMA guidelines to identify studies that evaluated the biomechanical properties of SA and TO techniques for repair of a ruptured QT. The search phrase used was “quadriceps tendon repair biomechanics”. Evaluated properties included ultimate load to failure (N), displacement (mm), stiffness (N/mm), and mode of failure.

**Results:**

Five studies met inclusion criteria, including a total of 72 specimens undergoing QT repair via the SA technique and 42 via the TO technique. Three of 4 studies found QTs repaired with SA to have significantly less elongation upon initial cyclic loading when compared to QTs repaired with the TO technique (*P* < .05). Three of 5 studies found QTs repaired with SA to have significantly less elongation upon final cyclic loading when compared to QTs repaired with the TO technique (*P* < .05). The pooled analysis from 4 studies reporting on initial displacement showed a statistically significant difference in favor of the SA group compared to the TO group (*P* = .03). The pooled analysis from studies reporting on secondary displacement and ultimate load to failure showed no significant difference between the SA and TO groups (*P* > .05). The most common mode of failure in both groups was suture slippage.

**Conclusion:**

On the basis of the included cadaveric studies, QTs repaired via the SA technique have less initial displacement upon cyclic testing when compared to QTs repaired via the TO technique. However, final displacement and ultimate load to failure outcomes did not reveal differences between the two fixation strategies. Knot slippage remains a common failure method for both strategies.

## Introduction

Quadriceps tendon (QT) ruptures are debilitating injuries that severely compromise knee function and ambulation.^1^^,^^2^ These injuries typically occur in patients older than 50 and while typically associated with either trauma or steroid use,^3^ they have also been associated with underlying medical conditions, including obesity, systemic illnesses, and renal dysfunction that may compromise the structural integrity of tendinous tissue.^2^^,^^4^^,^^5^ Overall, these injuries remain fairly uncommon, with an incidence of just over 1/100,000 patients per year.^4^ The mechanism of injury is a result of forceful eccentric loading about the knee joint with the foot planted.^3^ Excessive forces are borne by the musculotendinous complex, resulting in rupture that typically occurs at the superior patellar insertion.^4^

While various techniques for QT repair have been described, the two most popular methods for repair of a ruptured QT are the transosseous tunnel (TO) technique and the suture anchor (SA) technique. The TO technique uses parallel (typically 3) tunnels drilled in the midcoronal substance of the patella, aimed proximal to distal. The ruptured quadriceps tendon is typically debrided and sutured in a Krackow fashion with high-strength nonabsorbable sutures, followed by shuttling of sutures through the transosseous patellar bone tunnels, with the suture ends tied over the tunnels at the distal patella. This technique has a long track record of clinical success, and a low implant cost.^6^, ^7^, ^8^ Complications, while rare, include violation of either the articular surface or the superficial cortex of the patella with eccentric tunnel drilling, violation of the patellar tendon during retrieval of the sutures, alterations of extensor mechanism mechanics, and patellar fracture.^6^

Suture anchor fixation methods use a similar tendon-suturing strategy, with different bony fixation. Rather than drilling tunnels through the length of the patella, multiple anchors are inserted into the proximal pole of the patella, reducing the ruptured QT to the footprint on the patella. Anchor size and material can be variable, as can suture size and material. The theoretical advantages of this technique are lower risks for patellar fractures and cartilage injuries, which may be more likely to occur with errant tunnel placement in the TO technique.^9^ Suture anchor fixation has shortcomings as well, however. Implants typically cost more than suture alone, retained implants provide a potential nidus for infection, intra-articular violation is still possible, and the integrity of the repair construct is focused at the anchors. Although this technique has been associated with decreased operative times and requires decreased blood supply disruption,^10^ the reliance on the suture anchors for initial fixation strength has been a deterrent for many surgeons due to its increased complication rates.^11^

Multiple biomechanical studies^12^, ^13^, ^14^, ^15^, ^16^ comparing these two techniques for QT repair have been previously described; however, the biomechanical superiority of one technique over the other has yet to be investigated in a comprehensive review. The purpose of this study is to systematically review the literature to evaluate the biomechanical properties of the SA versus TO techniques for QT repair. The authors hypothesized that there would be no biomechanical differences between the SA and TO techniques.

## Materials and Methods

This systematic review was conducted according to PRISMA (Preferred Reporting Items for Systematic Reviews and Meta-Analyses) guidelines using a PRISMA checklist. Two independent reviewers (J.W.B. and A.L.) searched the PubMed, Embase, and Cochrane Library databases up to February 5, 2021. The electronic search strategy used was “quadriceps tendon repair biomechanics”*.* The inclusion criteria were human cadaveric studies that assessed the biomechanics of quadriceps tendon repair with a suture anchor and/or transosseous tunnel technique. Exclusion criteria included nonhuman cadaveric studies, studies that focused on repair of tendons other than the quadriceps tendon, nonbiomechanical studies, and studies without a full text available. Data extraction from each study was performed independently and then reviewed by a second author (J.W.B.). There was no need for funding or a third party to obtain any of the collected data.

The Quality Appraisal for Cadaveric Studies (QUACS) scale was used to evaluate cadaver study methodology quality.^18^ The scale consists of a checklist encompassing 13 items. Each is to be scored with either 0 (no/not stated) or 1 (yes/present) point. Points are only assigned if a criterion is met without any doubt, and a final percentage is given as the total score. Scores above 75% are considered satisfactory.

### Reporting Outcomes

All outcomes assessed were biomechanical in nature and included: ultimate load to failure (N), stiffness (N/mm), displacement (mm), and mode of failure. All included studies^12^, ^13^, ^14^, ^15^, ^16^ reported on displacement (mm), four studies^12^^,^^13^^,^^15^^,^^16^ reported on ultimate load to failure (N), four studies^12^^,^^13^^,^^15^^,^^16^ reported on mode of failure, and two studies^12^^,^^13^ reported on stiffness (N/mm).

### Statistical Analysis

When only standard errors were provided, standard deviations were calculated as described in the Cochrane Handbook for Systematic Reviews of Interventions (version 6.1.0).^17^ Multiple groups from the same study of identical size “*n*” with differing means and standard deviations were combined into a single group, according to the algorithm provided in the Cochrane Handbook for Systematic Reviews of Interventions (version 6.1.0).^17^ Weighted averages were calculated for all numerical outcomes when data from 3 or more studies were available. The outcomes were summarized in a forest plot when data from 3 or more studies were available. Using random-effects models, mean differences (MDs) with 95% confidence intervals (CIs) were calculated and included in the forest plot. A random-effects model was used because these models incorporate between-study heterogeneity into the overall summary measures. When there is no between-study heterogeneity, a random-effects model equals a fixed-effects model.^17^ In order to quantify the degree of heterogeneity due to between-study characteristics, *I*^*2*^ statistics were used to calculate heterogeneity. Meta-analyses statistics and generation of forest plots figures were performed using RevMan 5.3 (The Cochrane Collaboration, Copenhagen, Denmark).

## Results

**Fig 1:**
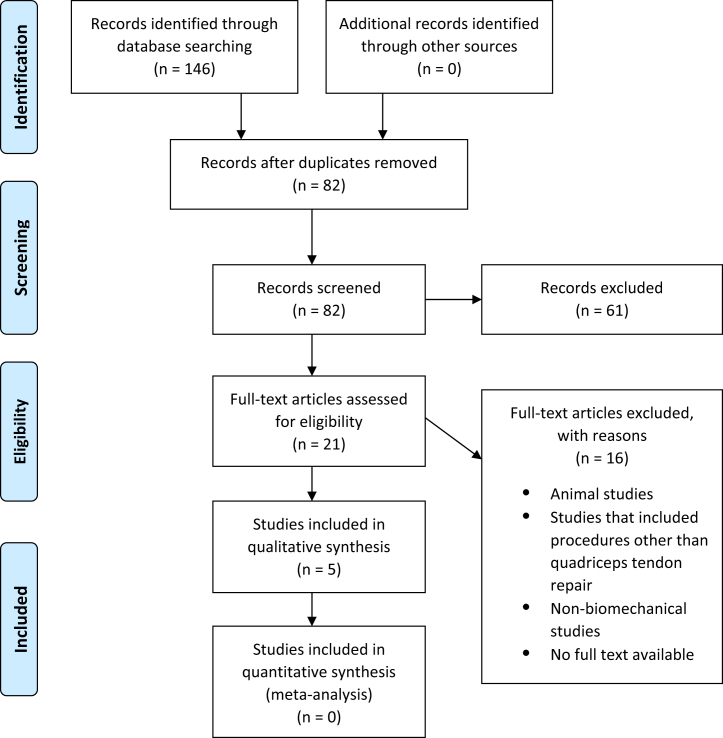
Preferred reporting items for systematic reviews and meta-analyses (PRISMA) flow diagram.

A total of 146 studies were reviewed by title and/or abstract to determine study eligibility based on inclusion criteria. Five studies, including a total of 114 cadaveric specimens undergoing QT repair (SA 72, TO 42), met inclusion and exclusion criteria and were included for analysis (Fig 1). These studies are summarized in Table 1.

**Table 1:** Studies Included and Outcomes Reported

Study	*n* (TO, SA)	Cadaver Age, years	Ultimate Load to Failure	Stiffness	Displacement	Mode of Failure
Hart et al., 2021^12^	5, 5	NR	+	+	+	+
Kindya et al., 2017^13^	10, 30	54.9 ± 13.7	+	+	+	+
Lighthart et al., 2008^14^	11, 11	NR	-	-	+	-
Petri et al., 2015^15^	10, 20	52.0 ± 13.0	+	-	+	+
Sherman et al., 2016^16^	6, 6	NR	+	-	+	+

Cadaver age is reported as means ± SD.

NR, not reported; SA, suture anchor, TO, transosseous tunnel. A “+” indicates that a study reported on a given outcome, while a “-” indicates that a study did not report on that outcome.

Study characteristics regarding number of tunnels, anchors, and companies used—Arthrex (Naples, FL) and Smith and Nephew (Tüttlingen, Germany)—are noted in Table 2. The sex for cadaveric specimens was not recorded in any study. All but one study^13^ used #2 sized suture in both the SA and TO constructs. Two studies^15^^,^^16^ recorded biomechanical data from an isolated extensor mechanism (i.e., patella and tendon only), while the remaining three studies^12^, ^13^, ^14^ created defects in the QT of an intact cadaveric knee.

**Table 2:** Summary of Repair Characteristics

Study	Techniques compared	Anchors Used	Number of Tunnels	Number of Anchors
Hart et al., 2021^12^	SA (Double Row), TO	Arthrex 5.5-mm single loaded Bio-Corkscrew FT (Proximal Row) Arthrex 3.5-mm bioabsorbable knotless PushLock (Distal Row)	3	4 (2 proximal, 2 distal)
Kindya et al., 2017^13^	SA, TO	Arthrex 5.5-mm single loaded (normal suture) Arthrex 4.75-mm biocomposite knotless SwiveLock (suture tape repair)	2	2
Lighthart et al., 2008^14^	SA, TO	Arthrex 5.5-mm single loaded	3	3
Petri et al., 2015^15^	SA (with Titanium or HA), TO	Smith and Nephew 5.5-mm double loaded	3	2 Titanium or 2 HA
Sherman et al., 2016^16^	SA, TO	Arthrex 4.5-mm Corkscrew, double loaded	3	3

Single and double loaded refer to the number of sutures initially loaded into the suture anchor. For one study,^15^ half of suture anchor repairs were done with normal suture, and half were done with suture tape.

HA, hydroxyapatite; NR, not reported; SA, suture anchor; TO, transosseous tunnel.

## Surgical Technique

### Suture Anchor

All five studies^12^, ^13^, ^14^, ^15^, ^16^ included cadavers undergoing QT repair with a SA technique. All included studies^12^, ^13^, ^14^, ^15^, ^16^ described debridement of the distal QT and proximal pole of the patella to allow for appropriate visualization of the anchor sites. Two or three guide holes were then drilled in either the medial and lateral thirds of the patella,^12^^,^^13^^,^^15^ or in all three thirds of the patella,^14^^,^^16^ respectively, depending on whether two or three anchors were used. One study^12^ described using two SAs at the proximal end of the patella in addition to the two anchors already placed at the patellar midpoint. One study^15^ divided their cadavers undergoing SA repair into two groups depending on SA material (titanium versus hydroxyapatite). After placing the anchors into the prepared sockets, all five studies^12^, ^13^, ^14^, ^15^, ^16^ described using stiches placed in Krackow fashion from distally to proximally through the tendon substance. One study^13^ described performing the SA technique with suture tape, in which a single, long suture was used to place a locking Krackow stitch in a distal to proximal and then proximal to distal direction within the QT, with tails exiting distally. Next, drill holes were made in the patella and knotless SAs were passed through each guide hole. The suture tape was then loaded within two knotless SAs and each anchor was malleted and screwed into place, thereby completing the repair. In the studies that evaluated mode of failure,^12^, ^13^, ^14^, ^15^, ^16^ suture tails were left intact to help identify suture slippage or knot failure during biomechanical testing.

### Transosseous Tunnel

All five studies^12^, ^13^, ^14^, ^15^, ^16^ included cadavers undergoing QT repair with a TO technique. All included studies^12^, ^13^, ^14^, ^15^, ^16^ described debridement of the distal QT and proximal pole of the patella followed by placement of standard locking stiches in Krackow fashion within the tendon substance from the distal to proximal and then proximal to distal direction. One study^13^ reported drilling two transpatellar tunnels that were placed in the medial and lateral thirds of the patella, while four studies^12^^,^^14^, ^15^, ^16^ reported drilling three transpatellar tunnels that were placed in all three thirds of the patella. Each end of the Krackow stitch was then shuttled through the tunnels from proximal to distal and then from distal to proximal. The repair was then cycled to remove creep from the system, and each strand was tied down to complete the repair.

### Methodologic Quality Assessment

The risk of bias and methodologic quality of the included studies were assessed using the QUACS scale,^18^ which has been previously validated (Table 3). The mean QUACS score was 84.6 ± 6.9 (range, 76.9-92.3). All five studies^12^, ^13^, ^14^, ^15^, ^16^ satisfied the threshold for a satisfactory level of methodologic quality (>75%).

**Table 3:** Quality Appraisal for Cadaveric Studies (QUACS)

Study	MCMS
Hart et al., 2021^12^	84.6
Kindya et al., 2017^13^	92.3
Lighthart et al., 2008^14^	76.9
Petri et al., 2015^15^	92.3
Sherman et al., 2016^16^	76.9
Total	84.6 ± 6.9

The “Total” row is reported as an average, with all values being reported as a percentage.

### Displacement

All included studies^12^, ^13^, ^14^, ^15^, ^16^ reported on displacement (mm). Three of four studies^13^, ^14^, ^15^, ^16^ reporting on initial displacement found QTs repaired with the SA technique to experience significantly less displacement after initial cyclic loading (measures displacement upon first cycles tested) when compared to QTs repaired with the TO technique (*P* < .05; Table 4). Similarly, three of the five studies^13^^,^^15^^,^^16^ reporting on final displacement found QTs repaired with the SA technique to experience significantly less displacement after final cyclic loading (measures displacement upon final cycles tested) when compared to QTs repaired with the TO technique (*P* < .05, Table 5).

**Table 4:** Initial Displacement (Ranged 10-100 Cycles)

Study	TO	SA	*P* Value
Kindya et al., 2017^13^	6.3 ± 1.9	3.9 ± 1.3	<0.05
Lighthart et al., 2008^14^	1.9 ± 1.5	2.4 ± 1.2	n.s.
Petri et al., 2015^15^	12.2 ± 3.2	3.6 ± 0.7	<0.05
Sherman et al., 2016^16^	4.7 ± 1.0	2.7 ± 0.5	<0.05
Total	6.3 ± 1.6	3.5 ± 0.9	0.03

Values are reported as a mean displacement (mm) ± SD. Initial displacement represents the measured elongation of the tendon after the first stage of cyclic loading. Initial loading cycles ranged from 10 to 100 cycles.

SA, suture anchor; TO, transosseous tunnel.

**Table 5:** Final Displacement (Ranged 130-1,000 Cycles)

Study	TO	SA	*P* Value
Hart et al., 2021^12^	8.0 ± 3.0	5.0 ± 4.0	n.s.
Kindya et al., 2017^13^	3.1 ± 0.9	2.1 ± 0.5	< 0.05
Lighthart et al., 2008^14^	4.5 ± 1.6	4.7 ± 1.5	n.s.
Petri et al., 2015^15^	33.3 ± 1.9	1.6 ± 0.5	< 0.05
Sherman et al., 2016^16^	9.1 ± 2.4	6.4 ± 1.3	< 0.05
Total	12.1 ± 2.2	2.9 ± 0.8	0.27

Values are reported as a mean displacement (mm) ± SD. Final displacement represents the measured elongation of the tendon after the final stage of cyclic loading. Final loading cycles ranged from 130 to 1,000 cycles.

n.s., nonsignificant; SA, suture anchor; TO, transosseous tunnel.

**Fig 2:**
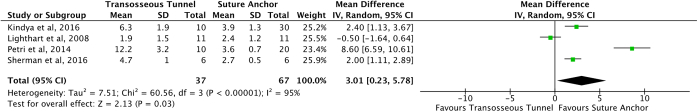
Forest plot of comparison of displacement initial (ranged 10-100 cycles) between transosseous tunnel and suture anchor techniques. CI, confidence interval, SD, standard deviation.

The pooled analysis from 4 studies^13^, ^14^, ^15^, ^16^ reporting on initial displacement showed a statistically significant difference in favor of the SA group (MD: 3.01 [95% CI: .23, 5.78]; *P* = .03) compared to the TO group (Fig 2). Statistical evaluation of heterogeneity found for initial displacement was *I*^*2*^ = 95% (*P* < .00001).

**Fig 3:**
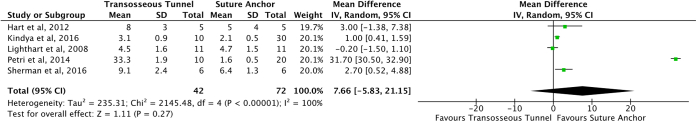
Forest plot of comparison of displacement secondary (ranged 130-1000 cycles) between transosseous tunnel and suture anchor techniques. CI, confidence interval; SD, standard deviation.

The pooled analysis from all 5 studies^12^, ^13^, ^14^, ^15^, ^16^ reporting on secondary displacement showed no significant difference between the SA and TO groups (MD: 7.66 [95% CI: −5.83, 21.15]; *P* = 0.27; Fig 3). Statistical evaluation of heterogeneity found for secondary displacement was *I*^*2*^ = 100% (*P* < .00001).

### Ultimate Load to Failure

Four studies^12^^,^^13^^,^^15^^,^^16^ reported on ultimate load to failure (Table 6). Two studies^13^^,^^15^ found the SA technique to a have significantly higher mean ultimate load to failure when compared to the TO technique (*P* < .05). Conversely, one study found the TO technique to have a significantly higher mean ultimate load to failure when compared to the SA technique (*P* = .04).

**Table 6:** Ultimate Load To Failure

Study	TO	SA	*P* Value
Hart et al., 2021^12^	591.0 ± 84.0	447.0 ± 86.0	0.04
Kindya et al., 2017^13^	413.0 ± 107.0	531.3 ± 153.9	< 0.05
Petri et al., 2015^15^	338.0 ± 60.0	656.0 ± 171.1	< 0.05
Sherman et al., 2016^16^	250.5 ± 42.0	286.0 ± 86.0	0.40
Total	386.1 ± 86.5	510.5 ± 127.6	0.28

All values are reported as a means ± SD, with the “Total” row reported as a weighted average.

SA, suture anchor; TO, transosseous tunnel.

**Fig 4:**
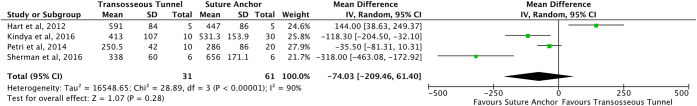
Forest plot of comparison of ultimate load to failure between transosseous tunnel and suture anchor techniques. CI, confidence interval, SD, standard deviation.

The pooled analysis from 4 studies^12^^,^^13^^,^^15^^,^^16^ reporting on ultimate load to failure showed no significant difference between the SA and TO groups (MD: −74.03 [95% CI: −209.46, 61.40]; *P* = .28; Fig 4). Statistical evaluation of heterogeneity found for ultimate load to failure was *I*^*2*^ = 90% (*P* < .00001).

### Stiffness

Two studies^12^^,^^13^ reported on stiffness (N/mm). One study^13^ found QTs repaired with the SA technique to experience significantly improved construct stiffness (52.5 ± 25.8 N/mm) compared to QTs repaired with the TO technique (26 ± 12 N/mm, *P* < .05). The other study^12^ found no differences between SA and TO groups.

### Mode of Failure

Four studies^12^^,^^13^^,^^15^^,^^16^ reported on mode of failure, with knot slippage being the most common for both the TO and SA techniques (Table 7).

**Table 7:** Mode of Failure

Failure Mode	Number of Instances
Transosseous Tunnel (*n* = 31)	
One knot slipped	12 (38.7%)
Suture tore through tendon	10 (32.3%)
Suture broke at knots/eyelet	9 (29.0%)
Suture Anchor (*n* = 61)	
One knot slipped	28 (45.9%)
Suture tore through tendon	14 (23.0%)
Suture broke at knots/eyelet	10 (16.4%)
Anchor pulled out from bone	7 (11.5%)
Other	2 (3.2%)

## Discussion

On the basis of the results of this systematic review, tendon displacement at initial cycling is consistently lower in SA fixation constructs when compared to constructs repaired with the TO technique. However, final displacement and ultimate load to failure outcomes did not reveal differences between the two fixation strategies. Similarly, there were unremarkable differences in construct stiffness and modes of failure between the SA and TO groups.

Acute tendon ruptures are typically the result of high eccentric loading, with forces borne by the enthesis, which is primarily made of type II collagen.^19^ Interestingly, quadriceps ruptures typically occur around the enthesis rather than the biomechanically weakest point: the myo-tendinous junction.^11^^,^^20^ Bone-tendon failure may be due to abnormal cellular structure at the rupture site. Kannus et al^21^ evaluated 891 spontaneous ruptures (82 quadriceps ruptures) and found abnormalities of hypoxic degenerative tendinopathy, mucoid degeneration, tendolipomatosis, and calcifying tendinopathy in nearly all biopsies taken at the time of repair. When considering the biomechanical differences between SA versus TO, differences in initial gapping may lead to decreased strength over time, as has been suggested in prior literature.^22^ Additionally, eccentric gapping has been shown to result from tendon repair in the Achilles,^23^ which may be exacerbated by the initial cycling load gaps seen in the TO group. To date, however, no human studies have evaluated the long-term biomechanical consequences of initial gap production differences among in vivo quadriceps tendon repairs. Unfortunately, failure rates and mechanisms at various stages of healing are difficult to evaluate, which demonstrates another shortcoming of cadaveric testing.

When evaluating failure type, both SA and TO constructs failed via knot slippage most commonly. Knot strength is directly related to knot material, surgeon experience,^24^ and the number and type of knots thrown.^25^ Although less common in the present review, one mode of failure that should be noted is the dislodged anchor. Suture anchor displacement is a complication unique to the SA technique and is a common concern for this construct. While this review demonstrates that suture dislodging is a relatively uncommon form of failure (occurring only 10.3% of the time, with failure at the eyelet being a common method of failure), this remains a considerable concern to many surgeons considering the construct. Similarly, while the comparison between titanium and hydroxyapatite screws yielded maximum load to failure data in favor of hydroxyapatite screws, the authors did note that failure of the eyelet was unique to that particular screw type.^15^ While a direct comparison between companies may be helpful, further studies directly comparing anchor diameter, number, and position may directly address many of the issues hindering widespread adaptation of the suture anchor technique.

Despite the included studies^12^, ^13^, ^14^, ^15^, ^16^ showing increased favorability for the SA technique in one biomechanical parameter (initial displacement), there is very limited clinical data to support superiority for either technique. While clinical outcomes are historically reported as good, these are largely based on the TO technique.^6^, ^7^, ^8^ Limited literature exists regarding the outcomes for SA fixation and is mostly limited to small case series.^26^^,^^27^ Bushnell et al.^28^ reported on 5 patients who underwent SA repair, 4 of whom had full return to activity. A small sample pilot study from Plesser et al.^29^ revealed statistically equivalent clinical outcomes and failure rates in 17 patients fixed with either TO or SA techniques. A single prospective multicenter study performed by Mille et al.^30^ evaluated 11 patients who underwent SA fixation of a QT rupture at a mean follow up of 14.7 months. The authors noted two retears (one of which was due to anchor displacement), with 82% of patients either satisfied or very satisfied. Further studies that prospectively evaluate clinical outcomes between these two surgical techniques are necessary to appropriately assess their impacts on clinical performance and determine whether or not these biomechanical findings are clinically relevant. Procedures performed in the lab can drastically differ from the same procedure performed in the operating room, and the results of any cadaveric study should be taken with caution. Although the current study demonstrates slight advantages of the SA technique over the TO technique, previously published clinical data on postoperative outcomes and complications should be prioritized when deciding which technique to use.

### Limitations

The limitations of this study should be noted. In particular, only five studies were included in this review. SA and TO surgical techniques were not identical across all studies, and there was variability in the way the reported biomechanical properties were tested (displacement, ultimate load to failure, stiffness), making direct comparison difficult. In addition, optimal anchor size could not be evaluated, as there was not enough data to perform a subanalysis on anchor size for the SA technique. Additionally, *I^2^* values for all three outcomes included in the meta-analysis suggested that considerable heterogeneity may be present, making it difficult to draw strong inferences from the available data due to underpowered statistics. Finally, not all studies reported on cadaver age or bone quality, which could have provided useful information on the quality of the tendinous tissue and the integrity of anchor fixation, respectively.

### Conclusion

On the basis of the included cadaveric studies, QTs repaired via the SA technique have less initial displacement upon cyclic testing when compared to QTs repaired via the TO technique. However, final displacement and ultimate load to failure outcomes did not reveal differences between the two fixation strategies. Knot slippage remains a common failure method for both strategies.

## Supplementary Data

ICMJE author disclosure forms
