# Supplementary material for: Biomechanical Testing of Suture Anchor Versus Transosseous Tunnel Technique for Quadriceps Tendon Repair Yields Similar Outcomes: A Systematic Review
Source: Arthrosc Sports Med Rehabil. 2021 Sep 30;3(6):e2059–66. doi: 10.1016/j.asmr.2021.08.013 (PMC8689238; doi:10.1016/j.asmr.2021.08.013)
Supplement: ICMJE author disclosure forms [file mmc1.pdf]

Arthroscopy: The Journal of Arthroscopic and Related Surgery

— Instructions —

ICMJE Form for Disclosure of Potential Conflicts of Interest

Each author of the manuscript must separately complete and save this form using his or her name in the file name. Each author's completed form must then be uploaded with the manuscript.

The purpose of this form is to provide readers of your manuscript with information about your other interests that could influence how they receive and understand your work. The form is in four parts:

Section 1. Identifying information

Enter your full name and provide the manuscript title.

Section 2. The work under consideration for publication

This section asks for information about the work that you have submitted for publication. The time frame for this reporting is that of the work itself, from the initial conception and planning to the present. The requested information is about resources that you received, either directly or indirectly (via your institution), to enable you to complete the work. Checking "No" means that you did the work without receiving any financial support from any third party — that is, the work was supported by funds from the same institution that pays your salary and that institution did not receive third-party funds with which to pay you. If you or your institution received funds from a third party to support the work, such as a government granting agency, charitable foundation, or commercial sponsor, check "Yes." Then complete the provide the information requested.

Section 3. Relevant financial activities outside the submitted work

This section asks about your financial relationships with entities in the biomedical arena that could be perceived to influence, or that give the appearance of potentially influencing, what you wrote in the submitted work. You should disclose interactions with ANY entity that could be considered broadly relevant to the work.

Report all sources of revenue paid (or promised to be paid) directly to you or your institution on your behalf over the 36 months prior to submission of the work. This should include all monies from sources with relevance to the submitted work, not just monies from the entity that sponsored the research. Please note that your interactions with the work's sponsor that are outside the submitted work should also be listed here. If there is any question, it is usually better to disclose a relationship than not to do so.

For grants you have received for work outside the submitted work, you should disclose support ONLY from entities that could be perceived to be affected financially by the published work, such as entities or foundations supported by entities that could be perceived to have a financial stake in the outcome. Public funding sources, such as government agencies, charitable foundations, or academic institutions, need not be disclosed. For example, if a government agency sponsored a study in which you have been involved and drugs were provided by a pharmaceutical company, you need only list the pharmaceutical company.

Section 4. Other relationships

Use this section to report other relationships or activities that readers could perceive to have influenced, or that give the appearance of potentially influencing, what you wrote in the submitted work.

ICMJE Form for Disclosure of Potential Conflicts of Interest

Section 1. Identifying Information

1. Given Name: John 2. Surname: Belk
3. Are you the corresponding author? Yes _X_ No__
4. Effective Date: 05/10/21
5. Manuscript Title: Suture Anchor Versus Transosseous Tunnel Technique for Quadriceps Tendon Repair: A Systematic Review of Biomechanical Strength

Section 2. The Work Under Consideration for Publication

Did you or your institution at any time receive payment or services from a third party for any aspect of the submitted work (including but not limited to grants, data monitoring board, study design, manuscript preparation, statistical analysis, etc…)?

Complete each item by typing an X in answer yes or not and completing the information requested if an answer is Yes. If you have more than one relationship, add lines.

1. Grant

_X_No ___Yes, money paid to you ___Yes, money paid to institution* Name of entity___ Comments†___

2. Consulting fee or honorarium

_X_No ___Yes, money paid to you ___Yes, money paid to institution* Name of entity___ Comments†___

3. Support for travel to meetings for the study or other purposes

_X_No ___Yes, money paid to you ___Yes, money paid to institution* Name of entity___ Comments†___

4. Fees for participation in review activities such as data monitoring boards, statistical analysis, end-point committees, and the like

_X_No ___Yes, money paid to you ___Yes, money paid to institution* Name of entity___ Comments†___

5. Payment for writing or reviewing the manuscript

_X_No ___Yes, money paid to you ___Yes, money paid to institution* Name of entity___ Comments†___

6. Provision of writing assistance, medicines, equipment, or administrative support

_X_No ___Yes, money paid to you ___Yes, money paid to institution* Name of entity___ Comments†___

7. Other

_X_No ___Yes, money paid to you ___Yes, money paid to institution* Name of entity___ Comments†___

* This means money that your institution received for your efforts on this study.
† Use this section to provide any needed explanation.

Section 3. Relevant financial activities outside the submitted work

1. Board membership

_X_No ___Yes, money paid to you ___Yes, money paid to institution* Name of entity___ Comments___

2. Consultancy

_X_No ___Yes, money paid to you ___Yes, money paid to institution* Name of entity___ Comments___

3. Employment

_X_No ___Yes, money paid to you ___Yes, money paid to institution* Name of entity___ Comments___

4. Expert testimony

_X_No ___Yes, money paid to you ___Yes, money paid to institution* Name of entity___ Comments___

5. Grants/grants pending

_X_No ___Yes, money paid to you ___Yes, money paid to institution* Name of entity___ Comments___

6. Payment for lectures including service on speakers bureaus

_X_No ___Yes, money paid to you ___Yes, money paid to institution* Name of entity___ Comments___

7. Payment for manuscript preparation

_X_No ___Yes, money paid to you ___Yes, money paid to institution* Name of entity___ Comments___

8. Patents (planned, pending or issued)

_X_No ___Yes, money paid to you ___Yes, money paid to institution* Name of entity___ Comments___

9. Royalties

_X_No ___Yes, money paid to you ___Yes, money paid to institution* Name of entity___ Comments___

10. Payment for development of educational presentations

_X_No ___Yes, money paid to you ___Yes, money paid to institution* Name of entity___ Comments___

11. Stock/stock options

_X_No ___Yes, money paid to you ___Yes, money paid to institution* Name of entity___ Comments___

12. Travel/accommodations/ meeting expenses unrelated to activities listed**

_X__No ___Yes, money paid to you ___Yes, money paid to institution* Name of entity___ Comments___

13. Other (err on the side of full disclosure)

_X_No ___Yes, money paid to you ___Yes, money paid to institution* Name of entity___ Comments___

** For example, if you report a consultancy above there is no need to report travel related to that consultancy on this line.

Section 4. Other relationships

Are there other relationships or activities that readers could perceive to have influenced, or that give the appearance of potentially influencing, what you wrote in the submitted work?

_X_ No other relationships/conditions/circumstances that present a potential conflict of interest

___Yes, the following relationships/conditions/circumstances are present (explain below):

>>>>>>>>>>><<<<<<<<<<<

The International Committee of Medical Journal Editors

The ICMJE Disclosure of Potential Conflicts of Interest Form was adopted by *Arthroscopy: The Journal of Arthroscopic and Related Surgery* along with 17 other leading orthopaedic journals at the 2011 annual meeting of the American Academy of Orthopaedic Surgeons.

Arthroscopy: The Journal of Arthroscopic and Related Surgery

— Instructions —

ICMJE Form for Disclosure of Potential Conflicts of Interest

Each author of the manuscript must separately complete and save this form using his or her name in the file name. Each author's completed form must then be uploaded with the manuscript.

The purpose of this form is to provide readers of your manuscript with information about your other interests that could influence how they receive and understand your work. The form is in four parts:

Section 1. Identifying information

Enter your full name and provide the manuscript title.

Section 2. The work under consideration for publication

This section asks for information about the work that you have submitted for publication. The time frame for this reporting is that of the work itself, from the initial conception and planning to the present. The requested information is about resources that you received, either directly or indirectly (via your institution), to enable you to complete the work. Checking "No" means that you did the work without receiving any financial support from any third party — that is, the work was supported by funds from the same institution that pays your salary and that institution did not receive third-party funds with which to pay you. If you or your institution received funds from a third party to support the work, such as a government granting agency, charitable foundation, or commercial sponsor, check "Yes." Then complete the provide the information requested.

Section 3. Relevant financial activities outside the submitted work

This section asks about your financial relationships with entities in the biomedical arena that could be perceived to influence, or that give the appearance of potentially influencing, what you wrote in the submitted work. You should disclose interactions with ANY entity that could be considered broadly relevant to the work.

Report all sources of revenue paid (or promised to be paid) directly to you or your institution on your behalf over the 36 months prior to submission of the work. This should include all monies from sources with relevance to the submitted work, not just monies from the entity that sponsored the research. Please note that your interactions with the work's sponsor that are outside the submitted work should also be listed here. If there is any question, it is usually better to disclose a relationship than not to do so.

For grants you have received for work outside the submitted work, you should disclose support ONLY from entities that could be perceived to be affected financially by the published work, such as entities or foundations supported by entities that could be perceived to have a financial stake in the outcome. Public funding sources, such as government agencies, charitable foundations, or academic institutions, need not be disclosed. For example, if a government agency sponsored a study in which you have been involved and drugs were provided by a pharmaceutical company, you need only list the pharmaceutical company.

Section 4. Other relationships

Use this section to report other relationships or activities that readers could perceive to have influenced, or that give the appearance of potentially influencing, what you wrote in the submitted work.

ICMJE Form for Disclosure of Potential Conflicts of Interest

Section 1. Identifying Information

1. Given Name: Adam 2. Surname: Lindsay
3. Are you the corresponding author? Yes __ No_X_
4. Effective Date: 05/10/21
5. Manuscript Title: Suture Anchor Versus Transosseous Tunnel Technique for Quadriceps Tendon Repair: A Systematic Review of Biomechanical Strength

Section 2. The Work Under Consideration for Publication

Did you or your institution at any time receive payment or services from a third party for any aspect of the submitted work (including but not limited to grants, data monitoring board, study design, manuscript preparation, statistical analysis, etc…)?

Complete each item by typing an X in answer yes or not and completing the information requested if an answer is Yes. If you have more than one relationship, add lines.

1. Grant

_X_No ___Yes, money paid to you ___Yes, money paid to institution* Name of entity___ Comments†___

2. Consulting fee or honorarium

_X_No ___Yes, money paid to you ___Yes, money paid to institution* Name of entity___ Comments†___

3. Support for travel to meetings for the study or other purposes

_X_No ___Yes, money paid to you ___Yes, money paid to institution* Name of entity___ Comments†___

4. Fees for participation in review activities such as data monitoring boards, statistical analysis, end-point committees, and the like

_X_No ___Yes, money paid to you ___Yes, money paid to institution* Name of entity___ Comments†___

5. Payment for writing or reviewing the manuscript

_X_No ___Yes, money paid to you ___Yes, money paid to institution* Name of entity___ Comments†___

6. Provision of writing assistance, medicines, equipment, or administrative support

_X_No ___Yes, money paid to you ___Yes, money paid to institution* Name of entity___ Comments†___

7. Other

_X_No ___Yes, money paid to you ___Yes, money paid to institution* Name of entity___ Comments†___

* This means money that your institution received for your efforts on this study.
† Use this section to provide any needed explanation.

Section 3. Relevant financial activities outside the submitted work

1. Board membership

_X__No __Yes, money paid to you ___Yes, money paid to institution* Name of entity___ Comments___

2. Consultancy

_X_No __Yes, money paid to you ___Yes, money paid to institution* Name of entity___ Comments___

3. Employment

_X_No ___Yes, money paid to you ___Yes, money paid to institution* Name of entity___ Comments___

4. Expert testimony

_X_No ___Yes, money paid to you ___Yes, money paid to institution* Name of entity___ Comments___

5. Grants/grants pending

_X_No ___Yes, money paid to you ___Yes, money paid to institution* Name of entity___ Comments___

6. Payment for lectures including service on speakers bureaus

_X_No ___Yes, money paid to you ___Yes, money paid to institution* Name of entity___ Comments___

7. Payment for manuscript preparation

_X_No ___Yes, money paid to you ___Yes, money paid to institution* Name of entity___ Comments___

8. Patents (planned, pending or issued)

_X_No ___Yes, money paid to you ___Yes, money paid to institution* Name of entity___ Comments___

9. Royalties

_X_No __Yes, money paid to you ___Yes, money paid to institution* Name of entity___ Comments___

10. Payment for development of educational presentations

_X_No ___Yes, money paid to you ___Yes, money paid to institution* Name of entity___ Comments___

11. Stock/stock options

_X_No ___Yes, money paid to you ___Yes, money paid to institution* Name of entity___ Comments___

12. Travel/accommodations/ meeting expenses unrelated to activities listed**

_X__No ___Yes, money paid to you ___Yes, money paid to institution* Name of entity___ Comments___

13. Other (err on the side of full disclosure)

_X_No ___Yes, money paid to you ___Yes, money paid to institution* Name of entity___ Comments___

** For example, if you report a consultancy above there is no need to report travel related to that consultancy on this line.

Section 4. Other relationships

Are there other relationships or activities that readers could perceive to have influenced, or that give the appearance of potentially influencing, what you wrote in the submitted work?

_X_No other relationships/conditions/circumstances that present a potential conflict of interest

___Yes, the following relationships/conditions/circumstances are present (explain below):

>>>>>>>>>>><<<<<<<<<<<

The International Committee of Medical Journal Editors

The ICMJE Disclosure of Potential Conflicts of Interest Form was adopted by *Arthroscopy: The Journal of Arthroscopic and Related Surgery* along with 17 other leading orthopaedic journals at the 2011 annual meeting of the American Academy of Orthopaedic Surgeons.

Arthroscopy: The Journal of Arthroscopic and Related Surgery

— Instructions —

ICMJE Form for Disclosure of Potential Conflicts of Interest

Each author of the manuscript must separately complete and save this form using his or her name in the file name. Each author's completed form must then be uploaded with the manuscript.

The purpose of this form is to provide readers of your manuscript with information about your other interests that could influence how they receive and understand your work. The form is in four parts:

Section 1. Identifying information

Enter your full name and provide the manuscript title.

Section 2. The work under consideration for publication

This section asks for information about the work that you have submitted for publication. The time frame for this reporting is that of the work itself, from the initial conception and planning to the present. The requested information is about resources that you received, either directly or indirectly (via your institution), to enable you to complete the work. Checking "No" means that you did the work without receiving any financial support from any third party — that is, the work was supported by funds from the same institution that pays your salary and that institution did not receive third-party funds with which to pay you. If you or your institution received funds from a third party to support the work, such as a government granting agency, charitable foundation, or commercial sponsor, check "Yes." Then complete the provide the information requested.

Section 3. Relevant financial activities outside the submitted work

This section asks about your financial relationships with entities in the biomedical arena that could be perceived to influence, or that give the appearance of potentially influencing, what you wrote in the submitted work. You should disclose interactions with ANY entity that could be considered broadly relevant to the work.

Report all sources of revenue paid (or promised to be paid) directly to you or your institution on your behalf over the 36 months prior to submission of the work. This should include all monies from sources with relevance to the submitted work, not just monies from the entity that sponsored the research. Please note that your interactions with the work's sponsor that are outside the submitted work should also be listed here. If there is any question, it is usually better to disclose a relationship than not to do so.

For grants you have received for work outside the submitted work, you should disclose support ONLY from entities that could be perceived to be affected financially by the published work, such as entities or foundations supported by entities that could be perceived to have a financial stake in the outcome. Public funding sources, such as government agencies, charitable foundations, or academic institutions, need not be disclosed. For example, if a government agency sponsored a study in which you have been involved and drugs were provided by a pharmaceutical company, you need only list the pharmaceutical company.

Section 4. Other relationships

Use this section to report other relationships or activities that readers could perceive to have influenced, or that give the appearance of potentially influencing, what you wrote in the submitted work.

ICMJE Form for Disclosure of Potential Conflicts of Interest

Section 1. Identifying Information

1. Given Name: Darby 2. Surname: Houck
3. Are you the corresponding author? Yes __ No_X_
4. Effective Date: 05/10/21
5. Manuscript Title: Suture Anchor Versus Transosseous Tunnel Technique for Quadriceps Tendon Repair: A Systematic Review of Biomechanical Strength

Section 2. The Work Under Consideration for Publication

Did you or your institution at any time receive payment or services from a third party for any aspect of the submitted work (including but not limited to grants, data monitoring board, study design, manuscript preparation, statistical analysis, etc…)?

Complete each item by typing an X in answer yes or not and completing the information requested if an answer is Yes. If you have more than one relationship, add lines.

1. Grant

_X_No ___Yes, money paid to you ___Yes, money paid to institution* Name of entity___ Comments†___

2. Consulting fee or honorarium

_X_No ___Yes, money paid to you ___Yes, money paid to institution* Name of entity___ Comments†___

3. Support for travel to meetings for the study or other purposes

_X_No ___Yes, money paid to you ___Yes, money paid to institution* Name of entity___ Comments†___

4. Fees for participation in review activities such as data monitoring boards, statistical analysis, end-point committees, and the like

_X_No ___Yes, money paid to you ___Yes, money paid to institution* Name of entity___ Comments†___

5. Payment for writing or reviewing the manuscript

_X_No ___Yes, money paid to you ___Yes, money paid to institution* Name of entity___ Comments†___

6. Provision of writing assistance, medicines, equipment, or administrative support

_X_No ___Yes, money paid to you ___Yes, money paid to institution* Name of entity___ Comments†___

7. Other

_X_No ___Yes, money paid to you ___Yes, money paid to institution* Name of entity___ Comments†___

* This means money that your institution received for your efforts on this study.
† Use this section to provide any needed explanation.

Section 3. Relevant financial activities outside the submitted work

1. Board membership

_X_No ___Yes, money paid to you ___Yes, money paid to institution* Name of entity___ Comments___

2. Consultancy

_X_No ___Yes, money paid to you ___Yes, money paid to institution* Name of entity___ Comments___

3. Employment

_X_No ___Yes, money paid to you ___Yes, money paid to institution* Name of entity___ Comments___

4. Expert testimony

_X_No ___Yes, money paid to you ___Yes, money paid to institution* Name of entity___ Comments___

5. Grants/grants pending

_X_No ___Yes, money paid to you ___Yes, money paid to institution* Name of entity___ Comments___

6. Payment for lectures including service on speakers bureaus

_X_No ___Yes, money paid to you ___Yes, money paid to institution* Name of entity___ Comments___

7. Payment for manuscript preparation

_X_No ___Yes, money paid to you ___Yes, money paid to institution* Name of entity___ Comments___

8. Patents (planned, pending or issued)

_X_No ___Yes, money paid to you ___Yes, money paid to institution* Name of entity___ Comments___

9. Royalties

_X_No ___Yes, money paid to you ___Yes, money paid to institution* Name of entity___ Comments___

10. Payment for development of educational presentations

_X_No ___Yes, money paid to you ___Yes, money paid to institution* Name of entity___ Comments___

11. Stock/stock options

_X_No ___Yes, money paid to you ___Yes, money paid to institution* Name of entity___ Comments___

12. Travel/accommodations/ meeting expenses unrelated to activities listed**

_X__No ___Yes, money paid to you ___Yes, money paid to institution* Name of entity___ Comments___

13. Other (err on the side of full disclosure)

_X_No ___Yes, money paid to you ___Yes, money paid to institution* Name of entity___ Comments___

** For example, if you report a consultancy above there is no need to report travel related to that consultancy on this line.

Section 4. Other relationships

Are there other relationships or activities that readers could perceive to have influenced, or that give the appearance of potentially influencing, what you wrote in the submitted work?

_X_No other relationships/conditions/circumstances that present a potential conflict of interest

___Yes, the following relationships/conditions/circumstances are present (explain below):

>>>>>>>>>>><<<<<<<<<<<

The International Committee of Medical Journal Editors

The ICMJE Disclosure of Potential Conflicts of Interest Form was adopted by *Arthroscopy: The Journal of Arthroscopic and Related Surgery* along with 17 other leading orthopaedic journals at the 2011 annual meeting of the American Academy of Orthopaedic Surgeons.

Arthroscopy: The Journal of Arthroscopic and Related Surgery

— Instructions —

ICMJE Form for Disclosure of Potential Conflicts of Interest

Each author of the manuscript must separately complete and save this form using his or her name in the file name. Each author's completed form must then be uploaded with the manuscript.

The purpose of this form is to provide readers of your manuscript with information about your other interests that could influence how they receive and understand your work. The form is in four parts:

Section 1. Identifying information

Enter your full name and provide the manuscript title.

Section 2. The work under consideration for publication

This section asks for information about the work that you have submitted for publication. The time frame for this reporting is that of the work itself, from the initial conception and planning to the present. The requested information is about resources that you received, either directly or indirectly (via your institution), to enable you to complete the work. Checking "No" means that you did the work without receiving any financial support from any third party — that is, the work was supported by funds from the same institution that pays your salary and that institution did not receive third-party funds with which to pay you. If you or your institution received funds from a third party to support the work, such as a government granting agency, charitable foundation, or commercial sponsor, check "Yes." Then complete the provide the information requested.

Section 3. Relevant financial activities outside the submitted work

This section asks about your financial relationships with entities in the biomedical arena that could be perceived to influence, or that give the appearance of potentially influencing, what you wrote in the submitted work. You should disclose interactions with ANY entity that could be considered broadly relevant to the work.

Report all sources of revenue paid (or promised to be paid) directly to you or your institution on your behalf over the 36 months prior to submission of the work. This should include all monies from sources with relevance to the submitted work, not just monies from the entity that sponsored the research. Please note that your interactions with the work's sponsor that are outside the submitted work should also be listed here. If there is any question, it is usually better to disclose a relationship than not to do so.

For grants you have received for work outside the submitted work, you should disclose support ONLY from entities that could be perceived to be affected financially by the published work, such as entities or foundations supported by entities that could be perceived to have a financial stake in the outcome. Public funding sources, such as government agencies, charitable foundations, or academic institutions, need not be disclosed. For example, if a government agency sponsored a study in which you have been involved and drugs were provided by a pharmaceutical company, you need only list the pharmaceutical company.

Section 4. Other relationships

Use this section to report other relationships or activities that readers could perceive to have influenced, or that give the appearance of potentially influencing, what you wrote in the submitted work.

ICMJE Form for Disclosure of Potential Conflicts of Interest

Section 1. Identifying Information

1. Given Name: Jason 2. Surname: Dragoo
3. Are you the corresponding author? Yes __ No_X_
4. Effective Date: 05/10/21
5. Manuscript Title: Suture Anchor Versus Transosseous Tunnel Technique for Quadriceps Tendon Repair: A Systematic Review of Biomechanical Strength

Section 2. The Work Under Consideration for Publication

Did you or your institution at any time receive payment or services from a third party for any aspect of the submitted work (including but not limited to grants, data monitoring board, study design, manuscript preparation, statistical analysis, etc…)?

Complete each item by typing an X in answer yes or not and completing the information requested if an answer is Yes. If you have more than one relationship, add lines.

1. Grant

_X_No ___Yes, money paid to you ___Yes, money paid to institution* Name of entity___ Comments†___

2. Consulting fee or honorarium

_X_No ___Yes, money paid to you ___Yes, money paid to institution* Name of entity___ Comments†___

3. Support for travel to meetings for the study or other purposes

_X_No ___Yes, money paid to you ___Yes, money paid to institution* Name of entity___ Comments†___

4. Fees for participation in review activities such as data monitoring boards, statistical analysis, end-point committees, and the like

_X_No ___Yes, money paid to you ___Yes, money paid to institution* Name of entity___ Comments†___

5. Payment for writing or reviewing the manuscript

_X_No ___Yes, money paid to you ___Yes, money paid to institution* Name of entity___ Comments†___

6. Provision of writing assistance, medicines, equipment, or administrative support

_X_No ___Yes, money paid to you ___Yes, money paid to institution* Name of entity___ Comments†___

7. Other

_X_No ___Yes, money paid to you ___Yes, money paid to institution* Name of entity___ Comments†___

* This means money that your institution received for your efforts on this study.
† Use this section to provide any needed explanation.

Section 3. Relevant financial activities outside the submitted work

1. Board membership

__No _X__Yes, money paid to you ___Yes, money paid to institution* Name of entity___ Comments___ AAOS, AJSM, AOSSM, AANA, PAC12 Athletic Conference

2. Consultancy

__No __X_Yes, money paid to you ___Yes, money paid to institution* Name of entity___ Comments___ Beckman Dickenson, Biomet, Breg, CONMED Linvatec, DePuy, DJ Orthopaedics, Genzyme, Harvest Technologies, Joint Restoration Foundation, KCRN Research, Ossur, Regeneration Technologies Inc, RNL Bio, Sideline Sports Docs, Zimmer

3. Employment

_X_No ___Yes, money paid to you ___Yes, money paid to institution* Name of entity___ Comments___

4. Expert testimony

_X_No ___Yes, money paid to you ___Yes, money paid to institution* Name of entity___ Comments___

5. Grants/grants pending

_X_No ___Yes, money paid to you ___Yes, money paid to institution* Name of entity___ Comments___

6. Payment for lectures including service on speakers bureaus

_X_No ___Yes, money paid to you ___Yes, money paid to institution* Name of entity___ Comments___

7. Payment for manuscript preparation

_X_No ___Yes, money paid to you ___Yes, money paid to institution* Name of entity___ Comments___

8. Patents (planned, pending or issued)

_X_No ___Yes, money paid to you ___Yes, money paid to institution* Name of entity___ Comments___

9. Royalties

_X_No ___Yes, money paid to you ___Yes, money paid to institution* Name of entity___ Comments___

10. Payment for development of educational presentations

_X_No ___Yes, money paid to you ___Yes, money paid to institution* Name of entity___ Comments___

11. Stock/stock options

_X_No ___Yes, money paid to you ___Yes, money paid to institution* Name of entity___ Comments___

12. Travel/accommodations/ meeting expenses unrelated to activities listed**

_X__No ___Yes, money paid to you ___Yes, money paid to institution* Name of entity___ Comments___

13. Other (err on the side of full disclosure)

_X_No ___Yes, money paid to you ___Yes, money paid to institution* Name of entity___ Comments___

** For example, if you report a consultancy above there is no need to report travel related to that consultancy on this line.

Section 4. Other relationships

Are there other relationships or activities that readers could perceive to have influenced, or that give the appearance of potentially influencing, what you wrote in the submitted work?

_X_No other relationships/conditions/circumstances that present a potential conflict of interest

___Yes, the following relationships/conditions/circumstances are present (explain below):

>>>>>>>>>>><<<<<<<<<<<

The International Committee of Medical Journal Editors

The ICMJE Disclosure of Potential Conflicts of Interest Form was adopted by *Arthroscopy: The Journal of Arthroscopic and Related Surgery* along with 17 other leading orthopaedic journals at the 2011 annual meeting of the American Academy of Orthopaedic Surgeons.

Arthroscopy: The Journal of Arthroscopic and Related Surgery

— Instructions —

ICMJE Form for Disclosure of Potential Conflicts of Interest

Each author of the manuscript must separately complete and save this form using his or her name in the file name. Each author's completed form must then be uploaded with the manuscript.

The purpose of this form is to provide readers of your manuscript with information about your other interests that could influence how they receive and understand your work. The form is in four parts:

Section 1. Identifying information

Enter your full name and provide the manuscript title.

Section 2. The work under consideration for publication

This section asks for information about the work that you have submitted for publication. The time frame for this reporting is that of the work itself, from the initial conception and planning to the present. The requested information is about resources that you received, either directly or indirectly (via your institution), to enable you to complete the work. Checking "No" means that you did the work without receiving any financial support from any third party — that is, the work was supported by funds from the same institution that pays your salary and that institution did not receive third-party funds with which to pay you. If you or your institution received funds from a third party to support the work, such as a government granting agency, charitable foundation, or commercial sponsor, check "Yes." Then complete the provide the information requested.

Section 3. Relevant financial activities outside the submitted work

This section asks about your financial relationships with entities in the biomedical arena that could be perceived to influence, or that give the appearance of potentially influencing, what you wrote in the submitted work. You should disclose interactions with ANY entity that could be considered broadly relevant to the work.

Report all sources of revenue paid (or promised to be paid) directly to you or your institution on your behalf over the 36 months prior to submission of the work. This should include all monies from sources with relevance to the submitted work, not just monies from the entity that sponsored the research. Please note that your interactions with the work's sponsor that are outside the submitted work should also be listed here. If there is any question, it is usually better to disclose a relationship than not to do so.

For grants you have received for work outside the submitted work, you should disclose support ONLY from entities that could be perceived to be affected financially by the published work, such as entities or foundations supported by entities that could be perceived to have a financial stake in the outcome. Public funding sources, such as government agencies, charitable foundations, or academic institutions, need not be disclosed. For example, if a government agency sponsored a study in which you have been involved and drugs were provided by a pharmaceutical company, you need only list the pharmaceutical company.

Section 4. Other relationships

Use this section to report other relationships or activities that readers could perceive to have influenced, or that give the appearance of potentially influencing, what you wrote in the submitted work.

ICMJE Form for Disclosure of Potential Conflicts of Interest

Section 1. Identifying Information

1. Given Name: James 2. Surname: Genuario
3. Are you the corresponding author? Yes __ No_X_
4. Effective Date: 05/10/21
5. Manuscript Title: Suture Anchor Versus Transosseous Tunnel Technique for Quadriceps Tendon Repair: A Systematic Review of Biomechanical Strength

Section 2. The Work Under Consideration for Publication

Did you or your institution at any time receive payment or services from a third party for any aspect of the submitted work (including but not limited to grants, data monitoring board, study design, manuscript preparation, statistical analysis, etc…)?

Complete each item by typing an X in answer yes or not and completing the information requested if an answer is Yes. If you have more than one relationship, add lines.

1. Grant

_X_No ___Yes, money paid to you ___Yes, money paid to institution* Name of entity___ Comments†___

2. Consulting fee or honorarium

_X_No ___Yes, money paid to you ___Yes, money paid to institution* Name of entity___ Comments†___

3. Support for travel to meetings for the study or other purposes

_X_No ___Yes, money paid to you ___Yes, money paid to institution* Name of entity___ Comments†___

4. Fees for participation in review activities such as data monitoring boards, statistical analysis, end-point committees, and the like

_X_No ___Yes, money paid to you ___Yes, money paid to institution* Name of entity___ Comments†___

5. Payment for writing or reviewing the manuscript

_X_No ___Yes, money paid to you ___Yes, money paid to institution* Name of entity___ Comments†___

6. Provision of writing assistance, medicines, equipment, or administrative support

_X_No ___Yes, money paid to you ___Yes, money paid to institution* Name of entity___ Comments†___

7. Other

_X_No ___Yes, money paid to you ___Yes, money paid to institution* Name of entity___ Comments†___

* This means money that your institution received for your efforts on this study.
† Use this section to provide any needed explanation.

Section 3. Relevant financial activities outside the submitted work

1. Board membership

_X__No ___Yes, money paid to you ___Yes, money paid to institution* Name of entity___ Comments___

2. Consultancy

__No _X__Yes, money paid to you ___Yes, money paid to institution* Name of entity___ Comments___ Pivot, Stryker

3. Employment

_X_No ___Yes, money paid to you ___Yes, money paid to institution* Name of entity___ Comments___

4. Expert testimony

_X_No ___Yes, money paid to you ___Yes, money paid to institution* Name of entity___ Comments___

5. Grants/grants pending

_X_No ___Yes, money paid to you ___Yes, money paid to institution* Name of entity___ Comments___

6. Payment for lectures including service on speakers bureaus

_X_No ___Yes, money paid to you ___Yes, money paid to institution* Name of entity___ Comments___

7. Payment for manuscript preparation

_X_No ___Yes, money paid to you ___Yes, money paid to institution* Name of entity___ Comments___

8. Patents (planned, pending or issued)

_X_No ___Yes, money paid to you ___Yes, money paid to institution* Name of entity___ Comments___

9. Royalties

__No ___Yes, money paid to you ___Yes, money paid to institution* Name of entity___ Comments___

10. Payment for development of educational presentations

_X_No ___Yes, money paid to you ___Yes, money paid to institution* Name of entity___ Comments___

11. Stock/stock options

_X_No ___Yes, money paid to you ___Yes, money paid to institution* Name of entity___ Comments___

12. Travel/accommodations/ meeting expenses unrelated to activities listed**

_X__No ___Yes, money paid to you ___Yes, money paid to institution* Name of entity___ Comments___

13. Other (err on the side of full disclosure)

_X_No ___Yes, money paid to you ___Yes, money paid to institution* Name of entity___ Comments___

** For example, if you report a consultancy above there is no need to report travel related to that consultancy on this line.

Section 4. Other relationships

Are there other relationships or activities that readers could perceive to have influenced, or that give the appearance of potentially influencing, what you wrote in the submitted work?

_X_ No other relationships/conditions/circumstances that present a potential conflict of interest

___Yes, the following relationships/conditions/circumstances are present (explain below):

>>>>>>>>>>><<<<<<<<<<<

The International Committee of Medical Journal Editors

The ICMJE Disclosure of Potential Conflicts of Interest Form was adopted by *Arthroscopy: The Journal of Arthroscopic and Related Surgery* along with 17 other leading orthopaedic journals at the 2011 annual meeting of the American Academy of Orthopaedic Surgeons.

— Instructions —

ICMJE Form for Disclosure of Potential Conflicts of Interest

Each author of the manuscript must separately complete and save this form using his or her name in the file name. Each author's completed form must then be uploaded with the manuscript.

The purpose of this form is to provide readers of your manuscript with information about your other interests that could influence how they receive and understand your work. The form is in four parts:

Section 1. Identifying information

Enter your full name and provide the manuscript title.

Section 2. The work under consideration for publication

This section asks for information about the work that you have submitted for publication. The time frame for this reporting is that of the work itself, from the initial conception and planning to the present. The requested information is about resources that you received, either directly or indirectly (via your institution), to enable you to complete the work. Checking "No" means that you did the work without receiving any financial support from any third party — that is, the work was supported by funds from the same institution that pays your salary and that institution did not receive third-party funds with which to pay you. If you or your institution received funds from a third party to support the work, such as a government granting agency, charitable foundation, or commercial sponsor, check "Yes." Then complete the provide the information requested.

Section 3. Relevant financial activities outside the submitted work

This section asks about your financial relationships with entities in the biomedical arena that could be perceived to influence, or that give the appearance of potentially influencing, what you wrote in the submitted work. You should disclose interactions with ANY entity that could be considered broadly relevant to the work.

Report all sources of revenue paid (or promised to be paid) directly to you or your institution on your behalf over the 36 months prior to submission of the work. This should include all monies from sources with relevance to the submitted work, not just monies from the entity that sponsored the research. Please note that your interactions with the work's sponsor that are outside the submitted work should also be listed here. If there is any question, it is usually better to disclose a relationship than not to do so.

For grants you have received for work outside the submitted work, you should disclose support ONLY from entities that could be perceived to be affected financially by the published work, such as entities or foundations supported by entities that could be perceived to have a financial stake in the outcome. Public funding sources, such as government agencies, charitable foundations, or academic institutions, need not be disclosed. For example, if a government agency sponsored a study in which you have been involved and drugs were provided by a pharmaceutical company, you need only list the pharmaceutical company.

Section 4. Other relationships

Use this section to report other relationships or activities that readers could perceive to have influenced, or that give the appearance of potentially influencing, what you wrote in the submitted work.

ICMJE Form for Disclosure of Potential Conflicts of Interest

Section 1. Identifying Information

1. Given Name: Stephanie 2. Surname: Mayer
3. Are you the corresponding author? Yes __ No_X_
4. Effective Date: 05/10/21
5. Manuscript Title: Suture Anchor Versus Transosseous Tunnel Technique for Quadriceps Tendon Repair: A Systematic Review of Biomechanical Strength

Section 2. The Work Under Consideration for Publication

Did you or your institution at any time receive payment or services from a third party for any aspect of the submitted work (including but not limited to grants, data monitoring board, study design, manuscript preparation, statistical analysis, etc…)?

Complete each item by typing an X in answer yes or not and completing the information requested if an answer is Yes. If you have more than one relationship, add lines.

1. Grant

_X_No ___Yes, money paid to you ___Yes, money paid to institution* Name of entity___ Comments†___

2. Consulting fee or honorarium

_X_No ___Yes, money paid to you ___Yes, money paid to institution* Name of entity___ Comments†___

3. Support for travel to meetings for the study or other purposes

_X_No ___Yes, money paid to you ___Yes, money paid to institution* Name of entity___ Comments†___

4. Fees for participation in review activities such as data monitoring boards, statistical analysis, end-point committees, and the like

_X_No ___Yes, money paid to you ___Yes, money paid to institution* Name of entity___ Comments†___

5. Payment for writing or reviewing the manuscript

_X_No ___Yes, money paid to you ___Yes, money paid to institution* Name of entity___ Comments†___

6. Provision of writing assistance, medicines, equipment, or administrative support

_X_No ___Yes, money paid to you ___Yes, money paid to institution* Name of entity___ Comments†___

7. Other

_X_No ___Yes, money paid to you ___Yes, money paid to institution* Name of entity___ Comments†___

* This means money that your institution received for your efforts on this study.
† Use this section to provide any needed explanation.

Section 3. Relevant financial activities outside the submitted work

1. Board membership

__X No __Yes, money paid to you ___Yes, money paid to institution* Name of entity___ Comments___

2. Consultancy

__No _X_Yes, money paid to you ___Yes, money paid to institution* Name of entity___ Comments___ Stryker

3. Employment

_X_No ___Yes, money paid to you ___Yes, money paid to institution* Name of entity___ Comments___

4. Expert testimony

_X_No ___Yes, money paid to you ___Yes, money paid to institution* Name of entity___ Comments___

5. Grants/grants pending

_X_No ___Yes, money paid to you ___Yes, money paid to institution* Name of entity___ Comments___

6. Payment for lectures including service on speakers bureaus

_X_No ___Yes, money paid to you ___Yes, money paid to institution* Name of entity___ Comments___

7. Payment for manuscript preparation

_X_No ___Yes, money paid to you ___Yes, money paid to institution* Name of entity___ Comments___

8. Patents (planned, pending or issued)

_X_No ___Yes, money paid to you ___Yes, money paid to institution* Name of entity___ Comments___

9. Royalties

_X_No __Yes, money paid to you ___Yes, money paid to institution* Name of entity___ Comments___

10. Payment for development of educational presentations

_X_No ___Yes, money paid to you ___Yes, money paid to institution* Name of entity___ Comments___

11. Stock/stock options

_X_No ___Yes, money paid to you ___Yes, money paid to institution* Name of entity___ Comments___

12. Travel/accommodations/ meeting expenses unrelated to activities listed**

_X__No ___Yes, money paid to you ___Yes, money paid to institution* Name of entity___ Comments___

13. Other (err on the side of full disclosure)

_X_No ___Yes, money paid to you ___Yes, money paid to institution* Name of entity___ Comments___

** For example, if you report a consultancy above there is no need to report travel related to that consultancy on this line.

Section 4. Other relationships

Are there other relationships or activities that readers could perceive to have influenced, or that give the appearance of potentially influencing, what you wrote in the submitted work?

_X_No other relationships/conditions/circumstances that present a potential conflict of interest

___Yes, the following relationships/conditions/circumstances are present (explain below):

>>>>>>>>>>><<<<<<<<<<<

The International Committee of Medical Journal Editors

The ICMJE Disclosure of Potential Conflicts of Interest Form was adopted by *Arthroscopy: The Journal of Arthroscopic and Related Surgery* along with 17 other leading orthopaedic journals at the 2011 annual meeting of the American Academy of Orthopaedic Surgeons.

— Instructions —

ICMJE Form for Disclosure of Potential Conflicts of Interest

Each author of the manuscript must separately complete and save this form using his or her name in the file name. Each author's completed form must then be uploaded with the manuscript.

The purpose of this form is to provide readers of your manuscript with information about your other interests that could influence how they receive and understand your work. The form is in four parts:

Section 1. Identifying information

Enter your full name and provide the manuscript title.

Section 2. The work under consideration for publication

This section asks for information about the work that you have submitted for publication. The time frame for this reporting is that of the work itself, from the initial conception and planning to the present. The requested information is about resources that you received, either directly or indirectly (via your institution), to enable you to complete the work. Checking "No" means that you did the work without receiving any financial support from any third party — that is, the work was supported by funds from the same institution that pays your salary and that institution did not receive third-party funds with which to pay you. If you or your institution received funds from a third party to support the work, such as a government granting agency, charitable foundation, or commercial sponsor, check "Yes." Then complete the provide the information requested.

Section 3. Relevant financial activities outside the submitted work

This section asks about your financial relationships with entities in the biomedical arena that could be perceived to influence, or that give the appearance of potentially influencing, what you wrote in the submitted work. You should disclose interactions with ANY entity that could be considered broadly relevant to the work.

Report all sources of revenue paid (or promised to be paid) directly to you or your institution on your behalf over the 36 months prior to submission of the work. This should include all monies from sources with relevance to the submitted work, not just monies from the entity that sponsored the research. Please note that your interactions with the work's sponsor that are outside the submitted work should also be listed here. If there is any question, it is usually better to disclose a relationship than not to do so.

For grants you have received for work outside the submitted work, you should disclose support ONLY from entities that could be perceived to be affected financially by the published work, such as entities or foundations supported by entities that could be perceived to have a financial stake in the outcome. Public funding sources, such as government agencies, charitable foundations, or academic institutions, need not be disclosed. For example, if a government agency sponsored a study in which you have been involved and drugs were provided by a pharmaceutical company, you need only list the pharmaceutical company.

Section 4. Other relationships

Use this section to report other relationships or activities that readers could perceive to have influenced, or that give the appearance of potentially influencing, what you wrote in the submitted work.

ICMJE Form for Disclosure of Potential Conflicts of Interest

Section 1. Identifying Information

1. Given Name: Rachel 2. Surname: Frank
3. Are you the corresponding author? Yes __ No_X_
4. Effective Date: 05/10/21
5. Manuscript Title: Suture Anchor Versus Transosseous Tunnel Technique for Quadriceps Tendon Repair: A Systematic Review of Biomechanical Strength

Section 2. The Work Under Consideration for Publication

Did you or your institution at any time receive payment or services from a third party for any aspect of the submitted work (including but not limited to grants, data monitoring board, study design, manuscript preparation, statistical analysis, etc…)?

Complete each item by typing an X in answer yes or not and completing the information requested if an answer is Yes. If you have more than one relationship, add lines.

1. Grant

_X_No ___Yes, money paid to you ___Yes, money paid to institution* Name of entity___ Comments†___

2. Consulting fee or honorarium

_X_No ___Yes, money paid to you ___Yes, money paid to institution* Name of entity___ Comments†___

3. Support for travel to meetings for the study or other purposes

_X_No ___Yes, money paid to you ___Yes, money paid to institution* Name of entity___ Comments†___

4. Fees for participation in review activities such as data monitoring boards, statistical analysis, end-point committees, and the like

_X_No ___Yes, money paid to you ___Yes, money paid to institution* Name of entity___ Comments†___

5. Payment for writing or reviewing the manuscript

_X_No ___Yes, money paid to you ___Yes, money paid to institution* Name of entity___ Comments†___

6. Provision of writing assistance, medicines, equipment, or administrative support

_X_No ___Yes, money paid to you ___Yes, money paid to institution* Name of entity___ Comments†___

7. Other

_X_No ___Yes, money paid to you ___Yes, money paid to institution* Name of entity___ Comments†___

* This means money that your institution received for your efforts on this study.
† Use this section to provide any needed explanation.

Section 3. Relevant financial activities outside the submitted work

1. Board membership

__No _X_Yes, money paid to you ___Yes, money paid to institution* Name of entity___ Comments___ AOSSM, ISAKOS, Orthopedics Today, AAOS, American Orthopaedic Society for Sports Medicine, American Shoulder and Elbow Surgeons, Arthroscopy Association of North America, International Cartilage Restoration Society, Journal of Shoulder and Elbow Surgery,

2. Consultancy

__No _X_Yes, money paid to you ___Yes, money paid to institution* Name of entity___ Comments___ Allosource, Arthrex, inc, JRF, Ossur

3. Employment

_X_No ___Yes, money paid to you ___Yes, money paid to institution* Name of entity___ Comments___

4. Expert testimony

_X_No ___Yes, money paid to you ___Yes, money paid to institution* Name of entity___ Comments___

5. Grants/grants pending

_X_No ___Yes, money paid to you ___Yes, money paid to institution* Name of entity___ Comments___

6. Payment for lectures including service on speakers bureaus

_X_No ___Yes, money paid to you ___Yes, money paid to institution* Name of entity___ Comments___

7. Payment for manuscript preparation

_X_No ___Yes, money paid to you ___Yes, money paid to institution* Name of entity___ Comments___

8. Patents (planned, pending or issued)

_X_No ___Yes, money paid to you ___Yes, money paid to institution* Name of entity___ Comments___

9. Royalties

_X_No __Yes, money paid to you ___Yes, money paid to institution* Name of entity___ Comments___

10. Payment for development of educational presentations

__No __X_Yes, money paid to you ___Yes, money paid to institution* Name of entity___ Comments___ Ossur, JRF, Arthrex Inc,

11. Stock/stock options

_X_No ___Yes, money paid to you ___Yes, money paid to institution* Name of entity___ Comments___

12. Travel/accommodations/ meeting expenses unrelated to activities listed**

_X__No ___Yes, money paid to you ___Yes, money paid to institution* Name of entity___ Comments___

13. Other (err on the side of full disclosure)

__No _X__Yes, money paid to you ___Yes, money paid to institution* Name of entity___ Comments___
Research Support from Smith & Nephew

** For example, if you report a consultancy above there is no need to report travel related to that consultancy on this line.

Section 4. Other relationships

Are there other relationships or activities that readers could perceive to have influenced, or that give the appearance of potentially influencing, what you wrote in the submitted work?

_X_No other relationships/conditions/circumstances that present a potential conflict of interest

___Yes, the following relationships/conditions/circumstances are present (explain below):

>>>>>>>>>>><<<<<<<<<<<

The International Committee of Medical Journal Editors

The ICMJE Disclosure of Potential Conflicts of Interest Form was adopted by *Arthroscopy: The Journal of Arthroscopic and Related Surgery* along with 17 other leading orthopaedic journals at the 2011 annual meeting of the American Academy of Orthopaedic Surgeons.

Arthroscopy: The Journal of Arthroscopic and Related Surgery

— Instructions —
ICMJE Form for Disclosure of Potential Conflicts of Interest

Each author of the manuscript must separately complete and save this form using his or her name in the file name. Each author's completed form must then be uploaded with the manuscript.

The purpose of this form is to provide readers of your manuscript with information about your other interests that could influence how they receive and understand your work. The form is in four parts:

Section 1. Identifying information

Enter your full name and provide the manuscript title.

Section 2. The work under consideration for publication

This section asks for information about the work that you have submitted for publication. The time frame for this reporting is that of the work itself, from the initial conception and planning to the present. The requested information is about resources that you received, either directly or indirectly (via your institution), to enable you to complete the work. Checking "No" means that you did the work without receiving any financial support from any third party — that is, the work was supported by funds from the same institution that pays your salary and that institution did not receive third-party funds with which to pay you. If you or your institution received funds from a third party to support the work, such as a government granting agency, charitable foundation, or commercial sponsor, check "Yes." Then complete the provide the information requested.

Section 3. Relevant financial activities outside the submitted work

This section asks about your financial relationships with entities in the biomedical arena that could be perceived to influence, or that give the appearance of potentially influencing, what you wrote in the submitted work. You should disclose interactions with ANY entity that could be considered broadly relevant to the work.

Report all sources of revenue paid (or promised to be paid) directly to you or your institution on your behalf over the 36 months prior to submission of the work. This should include all monies from sources with relevance to the submitted work, not just monies from the entity that sponsored the research. Please note that your interactions with the work's sponsor that are outside the submitted work should also be listed here. If there is any question, it is usually better to disclose a relationship than not to do so.

For grants you have received for work outside the submitted work, you should disclose support ONLY from entities that could be perceived to be affected financially by the published work, such as entities or foundations supported by entities that could be perceived to have a financial stake in the outcome. Public funding sources, such as government agencies, charitable foundations, or academic institutions, need not be disclosed. For example, if a government agency sponsored a study in which you have been involved and drugs were provided by a pharmaceutical company, you need only list the pharmaceutical company.

Section 4. Other relationships

Use this section to report other relationships or activities that readers could perceive to have influenced, or that give the appearance of potentially influencing, what you wrote in the submitted work.

ICMJE Form for Disclosure of Potential Conflicts of Interest

Section 1. Identifying Information

1. Given Name: Eric 2. Surname: McCarty
3. Are you the corresponding author? Yes __ No_X_
4. Effective Date: 05/10/21
5. Manuscript Title: Suture Anchor Versus Transosseous Tunnel Technique for Quadriceps Tendon Repair: A Systematic Review of Biomechanical Strength

Section 2. The Work Under Consideration for Publication

Did you or your institution at any time receive payment or services from a third party for any aspect of the submitted work (including but not limited to grants, data monitoring board, study design, manuscript preparation, statistical analysis, etc…)?

Complete each item by typing an X in answer yes or not and completing the information requested if an answer is Yes. If you have more than one relationship, add lines.

1. Grant

_X_No ___Yes, money paid to you ___Yes, money paid to institution* Name of entity___ Comments†___

2. Consulting fee or honorarium

_X_No ___Yes, money paid to you ___Yes, money paid to institution* Name of entity___ Comments†___

3. Support for travel to meetings for the study or other purposes

_X_No ___Yes, money paid to you ___Yes, money paid to institution* Name of entity___ Comments†___

4. Fees for participation in review activities such as data monitoring boards, statistical analysis, end-point committees, and the like

_X_No ___Yes, money paid to you ___Yes, money paid to institution* Name of entity___ Comments†___

5. Payment for writing or reviewing the manuscript

_X_No ___Yes, money paid to you ___Yes, money paid to institution* Name of entity___ Comments†___

6. Provision of writing assistance, medicines, equipment, or administrative support

_X_No ___Yes, money paid to you ___Yes, money paid to institution* Name of entity___ Comments†___

7. Other

_X_No ___Yes, money paid to you ___Yes, money paid to institution* Name of entity___ Comments†___

* This means money that your institution received for your efforts on this study.
† Use this section to provide any needed explanation.

Section 3. Relevant financial activities outside the submitted work

1. Board membership

__No _X_Yes, money paid to you ___Yes, money paid to institution* Name of entity___ Comments___
AOSSM, ISAKOS, Orthopedics Today, OrthopedicsAmerican Journal of Sports Medicine

2. Consultancy

__No _X_Yes, money paid to you ___Yes, money paid to institution* Name of entity___ Comments___
Biomet

3. Employment

_X_No ___Yes, money paid to you ___Yes, money paid to institution* Name of entity___ Comments___

4. Expert testimony

_X_No ___Yes, money paid to you ___Yes, money paid to institution* Name of entity___ Comments___

5. Grants/grants pending

_X_No ___Yes, money paid to you ___Yes, money paid to institution* Name of entity___ Comments___

6. Payment for lectures including service on speakers bureaus

_X_No ___Yes, money paid to you ___Yes, money paid to institution* Name of entity___ Comments___

7. Payment for manuscript preparation

_X_No ___Yes, money paid to you ___Yes, money paid to institution* Name of entity___ Comments___

8. Patents (planned, pending or issued)

_X_No ___Yes, money paid to you ___Yes, money paid to institution* Name of entity___ Comments___

9. Royalties

__No _X_Yes, money paid to you ___Yes, money paid to institution* Name of entity___ Comments___
Biomet, Elesevier, Zimmer

10. Payment for development of educational presentations

_X_No ___Yes, money paid to you ___Yes, money paid to institution* Name of entity___ Comments___

11. Stock/stock options

_X_No ___Yes, money paid to you ___Yes, money paid to institution* Name of entity___ Comments___

12. Travel/accommodations/ meeting expenses unrelated to activities listed**

_X__No ___Yes, money paid to you ___Yes, money paid to institution* Name of entity___ Comments___

13. Other (err on the side of full disclosure)

_X_No ___Yes, money paid to you ___Yes, money paid to institution* Name of entity___ Comments___

** For example, if you report a consultancy above there is no need to report travel related to that consultancy on this line.

Section 4. Other relationships

Are there other relationships or activities that readers could perceive to have influenced, or that give the appearance of potentially influencing, what you wrote in the submitted work?

_X_No other relationships/conditions/circumstances that present a potential conflict of interest

___Yes, the following relationships/conditions/circumstances are present (explain below):

>>>>>>>>>>><<<<<<<<<<<

The International Committee of Medical Journal Editors

The ICMJE Disclosure of Potential Conflicts of Interest Form was adopted by *Arthroscopy: The Journal of Arthroscopic and Related Surgery* along with 17 other leading orthopaedic journals at the 2011 annual meeting of the American Academy of Orthopaedic Surgeons.
